# Supplementary material for: Meta-evaluation of a whole systems programme, ActEarly: A study protocol
Source: PLoS One. 2023 Jun 1;18(6):e0280696. doi: 10.1371/journal.pone.0280696 (PMC10234514; doi:10.1371/journal.pone.0280696)

**Supporting material**

**S1 Figure.** Decision tree for the inclusion and exclusion of ActEarly activities in the meta-evaluation.


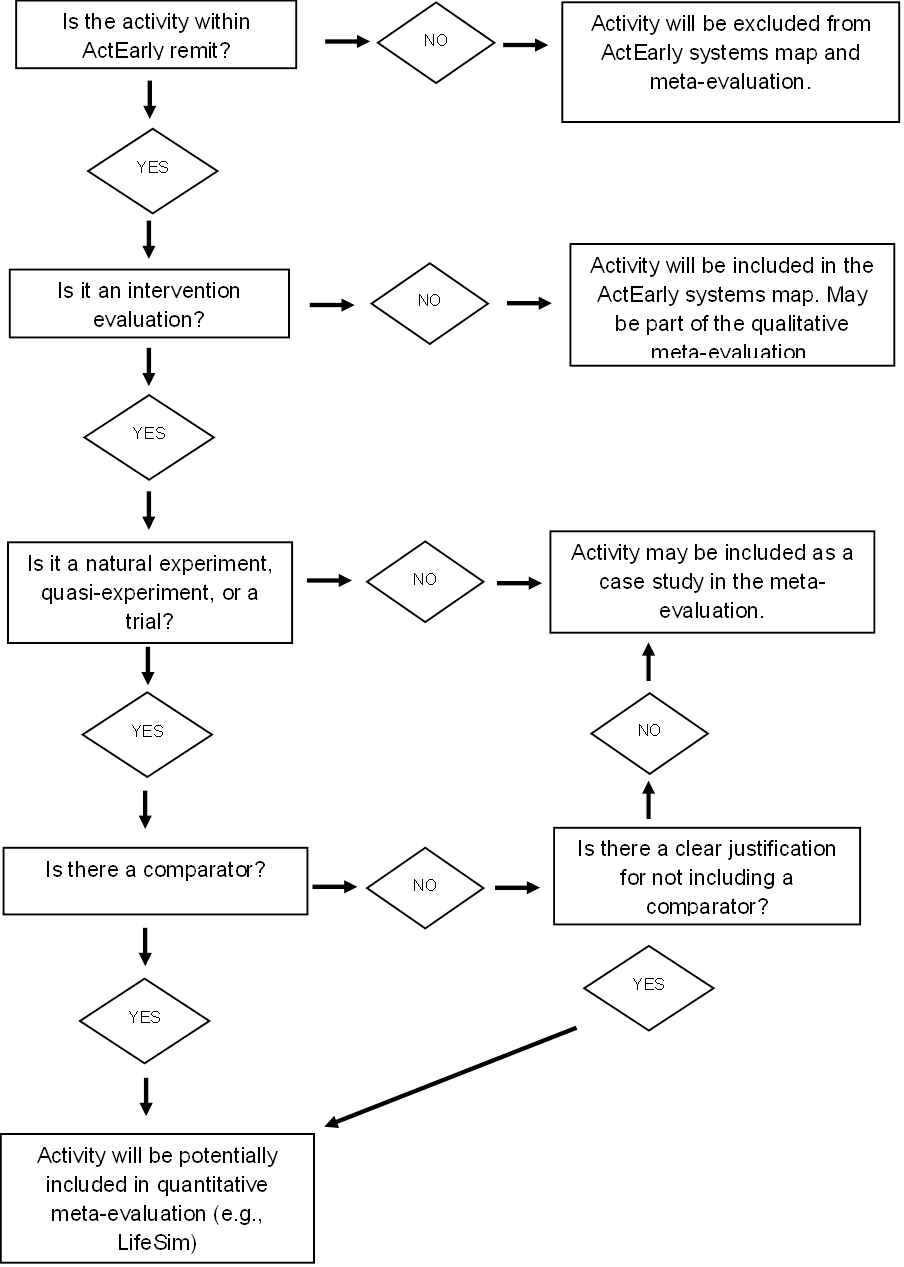

Supplement: S1 Fig — (DOC) [file pone.0280696.s001.doc]
